# Radiofrequency ablation of liver tumors: quantitative assessment of tumor coverage through CT image processing

**DOI:** 10.1186/1471-2342-13-3

**Published:** 2013-01-16

**Authors:** Katia Passera, Sabrina Selvaggi, Davide Scaramuzza, Francesco Garbagnati, Daniele Vergnaghi, Luca Mainardi

**Affiliations:** 1 Istituto di Ricerche Farmacologiche “Mario Negri” – IRCCS, Bergamo, Italy; 2 Dipartimento di Bioingegneria, Politecnico di Milano, Milan, Italy; 3 Fondazione IRCCS Istituto Nazionale dei Tumori, Milano, Italy

## Abstract

**Background:**

Radiofrequency ablation (RFA) is one of the most promising non-surgical treatments for hepatic tumors. The assessment of the therapeutic efficacy of RFA is usually obtained by visual comparison of pre- and post-treatment CT images, but no numerical quantification is performed.

**Methods:**

In this work, a novel method aiming at providing a more objective tool for the evaluation of RFA coverage is described. Image registration and segmentation techniques were applied to enable the visualization of the tumor and the corresponding post-RFA necrosis in the same framework. In addition, a set of numerical indexes describing tumor/necrosis overlap and their mutual position were computed.

**Results:**

After validation of segmentation step, the method was applied on a dataset composed by 10 tumors, suspected not to be completed treated. Numerical indexes showed that only two tumors were totally treated and the percentage of a residual tumor was in the range of 5.12%-35.92%.

**Conclusions:**

This work represents a first attempt to obtain a quantitative tool aimed to assess the accuracy of RFA treatment. The possibility to visualize the tumor and the correspondent post-RFA necrosis in the same framework and the definition of some synthetic numerical indexes could help clinicians in ameliorating RFA treatment.

## Background

Image-guided radiofrequency ablation is a powerful locoregional technique for the treatment of the non resectable primary and metastatic hepatic malignancies and a curative alternative to surgery in circumscribded tumors (inferior to 3 cm in diameter) [1]. Total necrotization of the target-tumor is the crucial condition of an effective treatment. However, there are many factors that could lead to an incomplete tumor ablation, such as insufficient visibility of tumor on US images, heat propagation prevented by blood vessels, and dimensions and morphology of the target tumor [2,3]. Therefore, it would be fundamental to assess the overlap between the tumor and the RFA necrotized tissues after the treatment. In current clinical procedures, the two major indicators of tumor necrotization and treatment efficacy are parenchyma changes seen on post-treatment CT and MR images and tumor recurrence in the patient’s follow-up. However, some subjectivity and uncertainty affect medical judgment about tumor coverage. The obstacles are: i) small contrast between necrosis and residual tumor, ii) inflammation process due to coagulative effects, iii) possible blood effusions [4].

Objective of this paper was to describe a method for quantitative assessment of RFA tumor coverage. To this purpose, we introduced a set of numerical indexes measuring overlap between tumor and RFA induced necrosis and their reciprocal position after realignment and segmentation of pre- and post-RFA CT images. In this work, segmentation of regions of interests (ROIs) was performed through a Fuzzy-C-means approach [5,6], while spatial correspondence between ROIs was obtained by realigning images through a non-linear B-splines-based algorithm [7,8] able to compensate for liver deformations.

## Methods

### Protocol

The method was tested in an experimental protocol developed at the Department of Images for Diagnosis and Therapy of the Fondazione IRCCS Istituto Nazionale dei Tumori (Milan, Italy) during 2007. 5 metastases and 5 hepatocellular carcinomas (HCCs) smaller than 20 cm^3^ (1-4 cm, tumor diameter range) were selected from 10 patients (5 men, 5 women; age range 40-86 years) treated with RFA as they were very complex cases not suitable for surgery. These tumors were selected among those suspected not to be completely treated. This was done in order to better evidence the potentiality of the proposed method.

All patients underwent intravenous multiphase dynamic CT before and after RFA. Pre-RFA CT scans were performed 5-10 days before RFA, while post-RFA scans were performed 15-20 days after the treatment.

#### Intravenous multiphase dynamic CT protocol

In this study, a Siemens SOMATOM Sensation 16 CT scanner (Erlangen, Germany) was used (gantry rotation speed of 420 ms, generator of 60 KW, pitch 1-1.5, 120 kVp, 250-300 mAs). 120–140 ml of nonionic Iopamiron 370 contrast agent were injected at a rate of 3 ml/s. Delay times after injection were 45, 80, and 180 seconds for the arterial, hepatic venous, and equilibrium phases, respectively. For HCC and hypervascular metastases arterial phase was considered, for hypovascular metastases portal or equilibrium phases were evaluated.

All CT scans were performed on inspiration. CT images were reconstructed on a 512x512 grid with a slice thickness of 5 mm and a pixel size of 0.74 mm (increment 0.6 mm, reconstruction 1-5 mm, medium smooth kernel (i30F), window/level 300/40).

#### RFA protocol

Depending on device availability, operator preference and tumor location, RFA was performed using a hook-tip needle housing 4 or 7 retractable curve electrodes (RITA Medical System, Mountain View, CA) or a 19 gauge MIRAS RC electrode (INVATEC, Brescia, Italia). The electrode was connected to a 460-KHz RF generator (Model 500 L; RITA Medical System, Mountain View, CA), which supplied a maximum power output of 110 W. In both cases, RFA was performed with real-time US guidance using a 3.5 MHz convex-probe (HDI 5000, ATL Ultrasound, Bathell, WA) and a guide device incorporated into the US probe in case of percutaneous puncture or during intraoperative approach. For all patients, the duration of RF energy application was in the range of 12-20 min. For HCC nodules, RFA procedure was performed after the interruption of their arterial supply by the occlusion of the hepatic artery with a balloon (diameter 11.5, length 2 cm) at the tip of a 7.0-F catheter (Medi-tech/Boston Scientific, Watertown, Mass). The occlusion balloon in the hepatic artery was filled with a mixture of saline solution and contrast materials.

RFA was performed by experienced physicians (70 RFA treatments per year).

### Image processing

In this work, the tumor coverage of RFA treatment was assessed by the integration between pre- and post-RFA CT images. Image processing was divided into three steps: (i) pre-processing (ii) segmentation and (iii) registration.

#### Pre-processing

**Figure 1:**
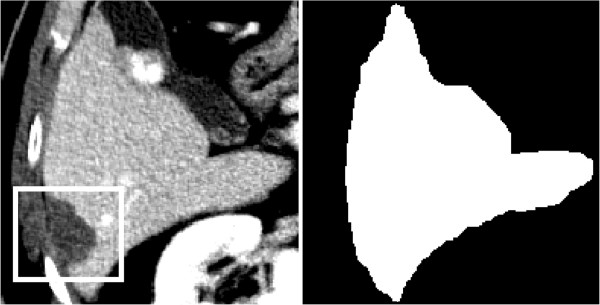
Peripheral necrosis on the liver edge, showing very low contrast with respect to the intercostal tissues (left) and correspondent liver binary mask obtained by Live-Wire technique (right).

To improve image quality and segmentation/registration performances the following pre-processing steps were applied:

• **image noise reduction.** CT image quality is often degraded by artifacts resulting from excessive X-ray quantum noise. In order to reduce image granularity and then to improve ROIs extraction, the original CT images were pre-filtered by a 5 × 5 median filter [9].

• **liver volume pre-segmentation.** Segmentation of tumors and necrosis on the liver edge was difficult because their typical gray levels were close to the intercostal tissues ones. In order to overcome this problem, a binary mask of the liver clearly separating liver from thorax pixels, was obtained [10,11] (Figure 1). To segment the liver region we used the Live–Wire algorithm [11] implemented in the MevisLab [12] software. Next, subsequent processing was applied to liver pixels only.

• **image contrast enhancement.** In order to obtain a sharp distinction among different clusters composing liver images, remapping of image dynamic range was applied. As liver intensity distribution is similar to the Gaussian distribution, it is possible to define the characteristic liver range analyzing the histogram of liver intensity into the volume pre-segmented by the Live–Wire technique. The lower and the higher gray levels that bounds the liver range were identified by two thresholds corresponding to the 2-3% of the liver histogram peak [13]. The defined liver range was then spread on the whole image dynamic range.

#### Segmentation

**Figure 2:**
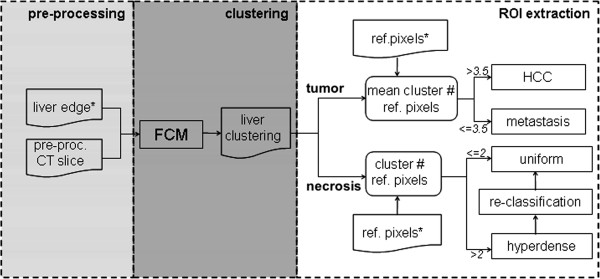
Steps of the segmentation algorithm ( * user interaction).

**Figure 3:**
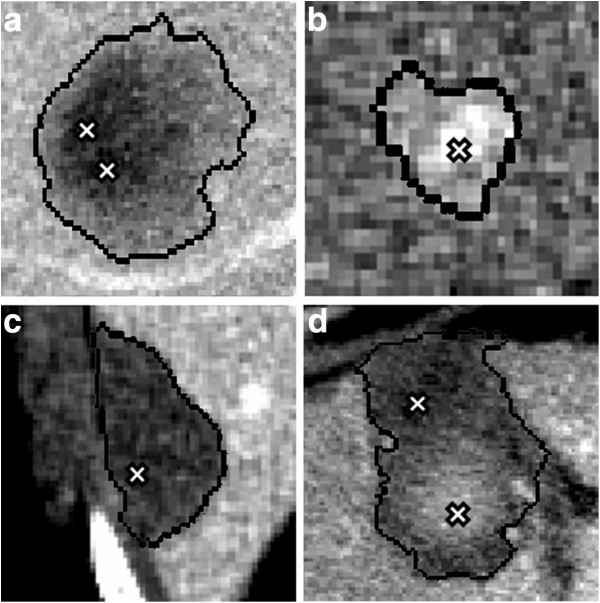
Examples of reference pixels on metastasis (a), HCC (b), uniform necrosis (c), and irregular necrosis with a hyperdense area due to some coagulative effects (d).

**Figure 4:**
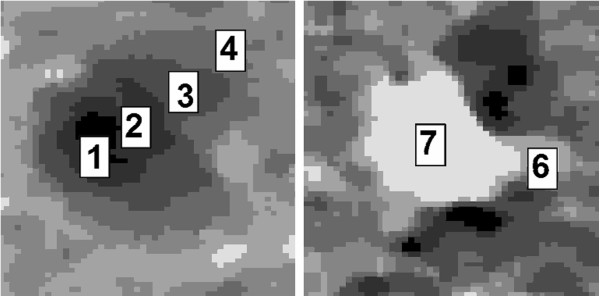
Examples of hepatic tumor partitioned in clusters: metastasis with a cluster index ranging from 1 to 4 (left) and small HCC with a cluster index ranging from 7 to 6 (right).

Figure 2 shows the main steps of our segmentation approach. The implemented algorithm is semi-automatic and gets as input the liver edge outlined by Live–Wire technique and a few reference pixels placed on tumor/necrosis by an operator through a graphical interface. Up to 3 pixels per region, representative of tumor/necrosis intensity may be selected among the darkest metastasis and uniform necrosis, or the brightest HCCs and hyperdense areas of irregular necrosis (Figure 3). Liver clustering was performed through a Fuzzy C-Means (FCM) algorithm [5,6] in order to cope with low contrast, intensity inhomogeneity and lack of clear edges that characterized both hepatic tumors and post-ablation necrosis [14,15]. After tuning of the method, the number of cluster was set at 7. Clusters were labeled according to their crescent centroid intensity (1 was the darkest, 7 the brightest). Once the image was segmented, we had to assign clusters to either tumors or necrosis. Since tumors and RFA necrosis showed different intensity patterns, the subsequent ROI extraction procedure was split into two different paths:

• **tumor.** Reference pixels were foremost used to define the range of tumor cluster indexes. Actually, the kind of tumors considered in this study (metastasis and small HCCs) was characterized by some geometric regularity. Tumors looked rather compact in the center, while they usually tended to vanish at the periphery. This means that, in the clusterized image, the tumor had a concentric ring structure. The mean of cluster indexes of reference pixels on all slices were used to identify the type of tumor: if it was inferior to 3.5 was a metastasis, while if superior, a HCC. Therefore, pixels were classified with increasing (for metastasis) or decreasing (for HCCs) cluster indexes from the center to the periphery of the tumor (Figure 4).

• **necrosis.** The implemented algorithm differentiated if the necrosis was uniformly hypodense or showed hyperdense areas due to some coagulative effects. In the clusterized image, uniformly hypodense necrosis were characterized by pixels belonging to the first two clusters corresponding to the darkest gray levels. These pixels were easily detected using the neighborhood conditions on the reference pixels picked by the operator. Instead, necrosis with hyperdense areas were identifiable by the presence of reference pixels with a cluster index greater than two. In the latter case, reference pixels were used to detect all hyperdense pixels and to further classify them iteratively with lower indexes. This classification allowed to treat irregular necrosis detection in the same way as uniform necrosis.

ROI extraction was particularly challenging for HCCs located very close to arterial vessels and for metastasis and necrosis on the liver edge. In these particular cases, morphological operators were used in order to distinguish real ROIs from those artificially extended due to partial volume effects at the liver periphery or to contrast enhanced opacified vessels.

The segmentation method was quite fast, taking about ten minutes considering user interaction in the live-wire technique and pixels picking.

#### Registration

Pre- and post-RFA images were registered through a B-spline free-form deformation algorithm [7,8], using the normalized mutual information (NMI) as similarity measure. Liver shifts and deformations were modeled by an affine global transformation and a local transformation based on a free-form deformation model of a regularly spaced control points grid. This registration technique had several advantages: it worked in the 3D space; it was efficient and robust, as the employed similarity measure (NMI) was not influenced by intensity changes in the processed images.

**Figure 5:**
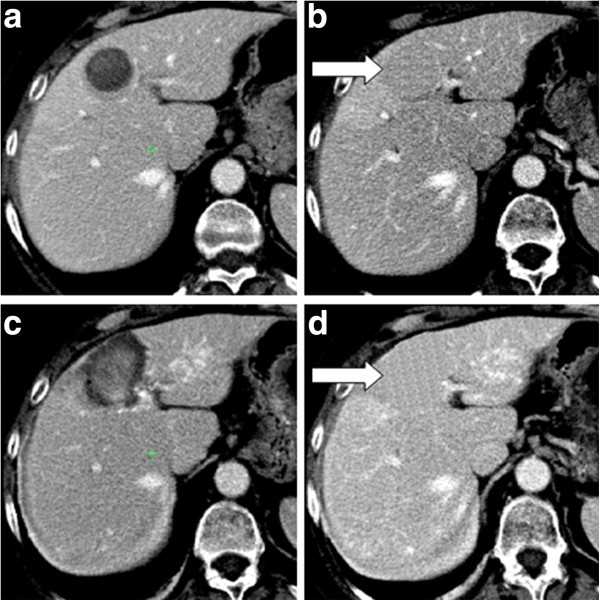
**An example of pre-RFA image with metastasis (a) and with synthetic pattern replacement of the metastasis (b).** Correspondent post-RFA image with necrosis (**c**) and with synthetic pattern replacement of the necrosis (**d**).

**Figure 6:**
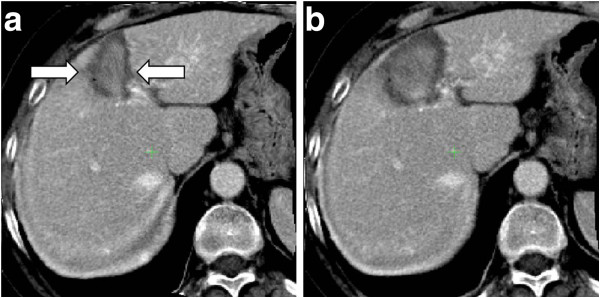
**The post-RFA image non-linearly registered without using synthetic pattern replacement (a) and using synthetic pattern (b).** The arrows show the effect of the fictitious deformations compensation.

However, an undesired compensation between tumor and necrosis might occur when performing pre- and post-RFA image registration, especially in the case of metastasis with gray levels very close to necrosis. In this situation, the registration method tended to overlap tumor and necrosis edges by modeling fictitious deformations. This effect was overcome by replacing, before image registration, the tumor ROI (in pre-RFA image) and the necrosis ROI (in post-RFA image) with a synthetic pattern (Figure 5), made out of a 19 × 19 pixels extracted from uniform liver parenchyma and replicated by radial padding. This new pair of images was then non–linearly registered and the output transformation was applied to the original images (Figure 6). The replacement of the original ROI with a synthetic pattern allowed to compensate for liver deformations, even if marked local deformations that might occur due to the coagulative process might not be corrected.

Registration step (fully automatic) took about 40 minutes. This time was evaluated on a PC (Intel Pentium III). The use of a more powerful or dedicated PC could improve this performance.

### Numerical Indexes for the RFA evaluation

**Figure 7:**
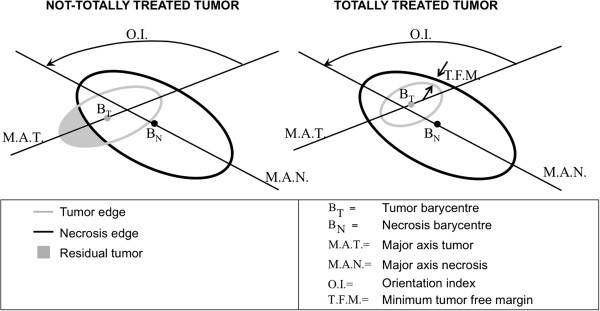
Numerical indexes used for RFA evaluation.

After having extracted and registered the pre-RFA tumor and the post-RFA necrosis, it was possible to measure their overlap and mutual position. As shown in Figure 7 there were two possible situations: a totally treated tumor or a not-totally treated tumor. To describe these situation four numerical indexes were defined:

• (i) **The residual tumor size**. All pixels belonging to the pre-RFA tumor and not included into necrosis area were labeled as not treated pixels. Then, the residual tumor size (given by the number of non treated pixels) was computed in volume and in percentage with respect to the pre-RFA tumor size.

• (ii) **The tumor free margin (T.F.M.)**. In the case of totally-treated tumor, it was possible to calculate the minimum T.F.M. by dilating pre-RFA tumor edge in a isotropic way until the tumor remains into the necrosis. The product between number of dilating iterations and pixel dimension gave the desired thickness. As the probability of tumor recidivism was higher for limited extra-tumor necrosis thickness [4], this index might have clinical relevance to indicate possible pathway for recidivism.

• (iii) **The inter-barycentric distance (|B**_**T**_**– B**_**N**_**|)**. This index computed the distance between tumor and necrosis barycenter (B_T_ and B_N_ , respectively) providing a measure of ROIs centering.

• (iv) **The orientation index (O.I.)**. For each slice, an ellipse was fitted to each ROI by making equal second order central moments of the ellipse to those of ROIs. Then, the angle (in degrees) between the major axis of the two ellipses was measured. This angle gave indication of the reciprocal orientation between tumor and necrosis ROIs and of potential favorite orientations for necrosis growth.

The inter-barycentric distance and the O.I. provided complementary elements for a more objective RFA evaluation. In fact, a not–totally treated tumor also resulted in a misalignment with the post-ablation necrosis. In particular, an inter-barycentric distance |B_T_ − B_N_ | with the same magnitude order of the target tumor was the natural consequence of an inaccurate tumor centering by the necrosis. Besides, an O.I. in the critical range 60° − 120° indicated an objective difficulty of the necrosis in following the tumor morphology and orientation.

### Segmentation validation

As segmentation was crucial in the proposed method, a detailed validation of this step was performed, following a classical approach reported in literature [16,17]. An experienced radiologist manually traced the contour of 10 tumors and 10 necrosis in our datasets. Manual contours were considered as Ground Truth (GT) and compared with the output ROIs obtained with the semi-automatic algorithm.

Pixels belonging to the ROI detected by the semi-automatic algorithm were classified as true positives (TP), false positives (FP), true negatives (TN) and false negatives (FN) on the basis of the comparison with the GT. Such assessment made possible the evaluation of the following indexes: Percentage Match (PM), Positive Predictivity (P+), Specificity (SPEC) and Negative Predictivity (P-) [18](see Table 1). PM index shows the correspondence between GT and algorithm segmentation. An ideal PM value was 100%, meaning that algorithm perfectly localized tumor/necrosis pixels. Conversely, the P+ index estimated the correspondence in size and location between the algorithm segmentation and GT. PM index did not take the potential error by excess into account; on the contrary P+ had low values if FP number was high because of an error by excess. A good segmentation thus required high values for both PM and P+.

**Table 1:** The numerical indexes used in the evaluation of segmentation performance

**Segmentation validation indexes [%]**	
**Percentage Match**	$\frac{\mathrm{TP}}{\mathrm{TP}+\mathrm{FN}}.100$
**Positive Predictivity**	$\frac{\mathrm{TP}}{\mathrm{TP}+\mathrm{FP}}.100$
**Specificity**	$\frac{\mathrm{TN}}{\mathrm{TP}+\mathrm{FP}}.100$
**Negative Predictivity**	$\frac{\mathrm{TN}}{\mathrm{TN}+\mathrm{FN}}.100$

## Results

The proposed method was applied to 8 out of 10 available cases as for 2 patients it was not possible to obtain a satisfactory realignment due to contingent image features: in the first case, serous fluid effusion in the abdominal cavity (ascites) caused the presence of a hypodense strip in the post–RFA image only; in the second one, the patient underwent not only RFA-treatment but also hepatic resection. In both cases the registration algorithm was not able to correct differences between pre- and post-RFA images. Such cases were thus used for the segmentation validation only. In the remaining 8 cases (4 HCCs and 4 metastasis), the complete RFA assessment procedure was performed.

### Segmentation validation

**Figure 8:**
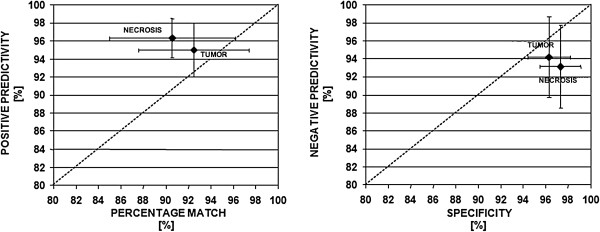
Mean and standard deviation of Percentage Match (PM) vs Positive Predictivity (P+) (left) and of Specificity (SPEC) vs Negative Predictivity (P-) (right).

Figure 8(left) shows trade-off PM vs. P+. PM and P+ mean values are very close to the optimum point on the top-right (PM > 90% and P + > 94%) and, in addition, the necrosis PM mean value is lower than the tumors one. This is a consequence of an important difference between tumors and necrosis. Although they both had vanishing edges, the segmentation algorithm had to extract the whole area including vanishing parts only for tumors; for necrosis, only the most compact area of the ROI was extracted because the peripheral vanishing parts were considered not perfectly treated and then exposed to the tumor recidivism risk.

The implemented segmentation algorithm took these considerations into account. In fact, for tumors the whole cluster range was assessed starting from the operator reference pixels, while for necrosis only the first two clusters (corresponding to the darkest and the most compact ROI area) were extracted, limiting risk of error by excess in necrosis segmentation procedure.

In order to assess the opposite risk of error by defect SPEC and P- indexes were calculated. Figure 8(right) shows that also the SPEC and P- mean values are very close to the optimum point on the top-right angle (SPEC > 96% and P− > 92%). This demonstrates a good balance between errors by excess and by defect in the segmentation process.

### Assessment of RFA tumor coverage

The pre-RFA and post-RFA image registration and segmentation operations enabled to visualize in the same framework the overlap between the pre-RFA tumor and the corresponding necrosis. In this way, information was automatically integrated and it was not required a physician’s effort in finding anatomical markers for visually locating the tumor after the RFA-treatment.

**Figure 9:**
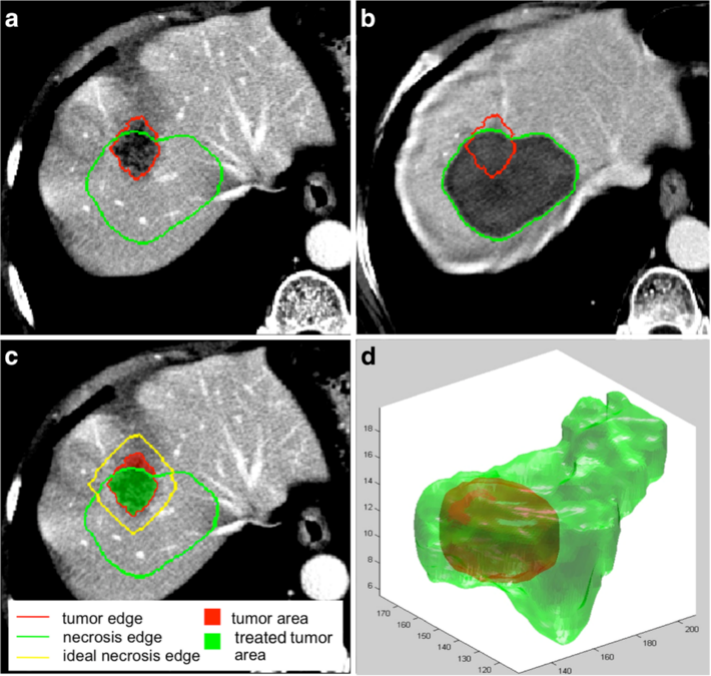
Different way to visualize tumor and necrosis in RFA treatment: pre-RFA image with enhanced tumor and necrosis edges(a), correspondent post-RFA image with enhanced tumor and necrosis edges (b), pre-RFA image with enhanced tumor treated and not-treated areas and the ideal necrosis edge (c), 3D render of a tumor and correspondent necrosis (d).

Figure 9 shows different ways of integrating information, either by visualizing slice-by-slice tumor and necrosis edges on both pre-RFA (Figure 9(a)) and post-RFA (Figure 9(b)) images or by distinguishing the treated from the not-treated tumor area and comparing the necrosis edge with respect to an ideal one (1 cm thick) (Figure 9(c)). Finally, tumor and necrosis volume rendering provided an easier understanding of the relationship between the tumor and ablation zone in the 3D space (Figure 9(d)), which would have been hard to analyze by a simple visual inspection due to the presence, on the post-RFA image, of some hypovascularised areas close to the original metastasis. In addition, the proposed method provided a set of numerical indexes able to quantify the overlap between pre-RFA tumor and post-RFA necrosis ROIs. Table 2 reports results of the quantitative analysis for the 4 metastasis and 4 HCCs (tumor volume range, 1.5 – 19.4 cm^3^). The proposed method classified only two tumors as totally treated, the percentage of residual post-RFA tumor being in the range of 5.12%-35.92%. Indexes values were computed slice-by-slice and results were summarized by using mean and standard deviation or min-max range of values. In all analyzed cases, T.F.M. was not homogeneous in all directions. Maximum and minimum T.F.M., computed for all tumors including those classified as not–totally treated (minimum T.F.M. set to 0 in slices in which tumor was not–totally treated), resulted to be always less than 4 mm (except for Case4 in which maximum T.F.M was 6.68 mm).

**Table 2:** The quantitative analysis of the tumor and post-ablation necrosis overlap for 8 cases

	**Tumor slices (#)**	**Tumor volume (#)**	Residual tumor* (cm3) [%]	**T.F.M. (mm) [min max]**	**|B**_**T**_**-B**_**N**_**| (mm) [mean ± std]**	**O.I. (degree) [mean ± std]**
Case 1 **META**	5	6.90	0.58 [8.48%]	0 - 1.73	11.59 ± 1.52	57.46 ± 22.22
Case 2 **META**	8	19.38	0 [0%]	0.74 - 2.23	4.96 ± 3.54	33.59 ± 20.82
Case 3 **META**	4	4.53	0.77 [17.01%]	0 - 2.16	7.60 ± 0.45	74.72 ± 5.27
Case 4 **META**	7	14.48	1.42 [9.83%]	0 - 6.69	16.98 ± 3.38	56.60 ± 18.54
Case 5 **HCC**	5	3.88	0 [0%]	1.93 - 3.87	6.61 ± 1.52	63.94 ± 4.50
Case 6 **HCC**	3	2.20	1.41 [35.92%]	0	4.07 ± 0.17	25.31 ± 11.03
Case 7 **HCC**	7	16.4	0.84 [5.12%]	0 - 3.64	7.57 ± 3.30	19.04 ± 12.75
Case 8 **HCC**	2	1.53	0.11 [7.01%]	0	2.75 ± 2.41	31.33 ± 20.08

*The residual tumor volume after RFA treatment.

**Figure 10:**
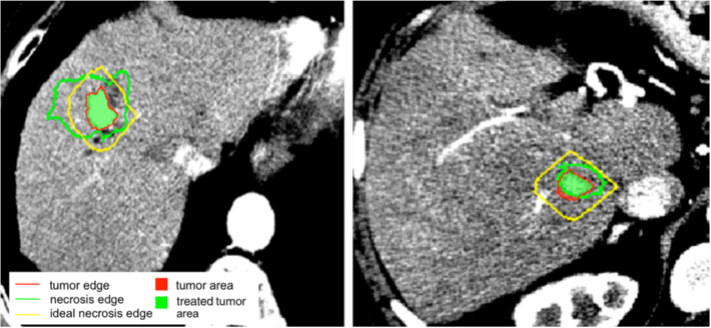
**A totally treated tumor (a) and a not-totally treated tumor (b).** Tumor and necrosis are visualized as in Figure 9 (**c**).

We show example of two opposite cases: a totally treated tumor (Figure 10(a),Case5) and the incomplete tumor treatment (Figure 10(b), Case6). In the former case, O.I. was about 65° and the T.F.M. < 2 mm, highlighting the necrosis difficulty in expanding along the anterior-posterior direction; in the latter the null T.F.M. confirmed the non-tumor coverage of the treatment despite the good parallelism between tumor and necrosis (O.I. ≃ 25°).

## Discussion and conclusion

In this paper, we presented a novel method to assess the hepatic tumor coverage of RFA. In the following, some comments about the method, the results and the study limitations were reported.

### Methodologies

A previous study [19] dealt with the problem of finding an objective RFA assessment method based on CT images segmentation and registration. There were three main differences between that study and the method proposed in this work. First of all, the method proposed in [19] was designed for pre- and post-RFA images fusion in order to enable easier understanding of the relationship between the tumor and ablation zone and to help to judge whether an ablative margin was ensured or not, but did not provide any numeric index for quantitative analysis and assessment of the RFA treatment. Secondly, in [19] ROI segmentation was performed manually, while in this work was based on a semi-automatic segmentation algorithm, requiring user interaction only for the selection of a few reference pixels needed to outline liver edges by the Live–Wire technique. As compared with manual segmentation, the algorithm used in this work was more efficient, as it was based on the automatic pixels gray level analysis performed through a Fuzzy-C-Means approach, it was reproducible thus avoiding uncertainty and subjectivity of manual edges outlining.

Finally, in our method, the pre- and post-RFA CT image realignment was based on the B-splines free form deformation algorithm [7,8] that allowed a non-linear hepatic volumes registration, instead of a simpler rigid registration algorithm with additional manual adjustments, which compensated only for translation and rotation shifts that occurred between pre- and post-RFA acquisitions. As the liver is a soft tissue undergoing non-linear deformations, mainly caused by respiration, heart pulsations and adjacent organ movements, a non-linear registration is required for correct volumes realignment [20,21].

There were peculiar properties which made the proposed algorithm particularly appealing for the assessment of RFA treatment in liver tumors: the short interaction time required by users to outline liver edges by the Live–Wire technique and to acquire the reference pixels on the tumor and necrosis ROIs (a few minutes for each image volume), and the accuracy, proved by good results of segmentation validation.

### Results

Even if in this study the tumor coverage of RFA was assessed on a small dataset of suspected non-completely treated tumors, it is worth noting that most of the tumors were not totally-treated (6 out of 8) and the percentage of a residual tumor was in the range of 5.12%-35.92%. In addition, T.F.M. was always well far from 1 cm-thick as recommended in current RFA guidelines [4].

There are several reasons that may explain these results. Before the treatment, the surgeon might be often aware that a large T.F.M. was not possible. In particular, the target tumor was frequently close to vital anatomical structures which had to be prevented from heat injury. Besides, there were physical limitations to heat spread associated to the liver blood flow (heat loss due to convection), that were enhanced also by O.I. and inter-barycentric distance indexes. Finally, the type of RFA treatment might affect the heat spread, such as in the case of RFA-treatment executed after occlusion of tumor blood supply. It was demonstrated that a larger necrosis area can be created when RFA treatment is performed in HCC nodules after their arterial supply occlusion [22-24]. In fact, there is a different temperature distribution within and around the HCC nodule and this phenomenon seems related to the difference in vascularization between HCC and the surrounding cirrhotic hepatic tissue [23]. The latter has a dual blood supply and is nourished mainly by the portal vein, which provides about two-thirds of the blood flow. HCC, however, is nourished mainly by the hepatic artery, with the portal vein providing a minor blood supply and the main venous drainage. Therefore acute occlusion of arterial flow is soon followed by a decrease in pressure within the HCC nodule, which continues to be perfused by means of reversed portal flow and, in some cases, by small collateral arteries.Thus, although the blood flow supplying the HCC nodule is substantially impaired, changing from high to sluggish flow, the blood flow supplying the surrounding hepatic tissue is only marginally modified. This results in an almost complete lack of heat loss due to convection within the HCC nodule, whereas intact and perhaps even increased portal blood flow in the surrounding tissue acts as an efficient heat sink that prevents heat diffusion outside the HCC nodule [23]. Therefore, the resultant necrosis reproduces the shape of HCC nodules and spare surrounding non-tumor tissues, producing a really tiny T.F.M.

### Study limitations

The present study had some limitations and some improvements could be performed. First of all, a larger data-set for the quantitative analysis of RFA-treatment could allow an adequately statistical analysis of the results. In addition, the evaluation of segmentation was performed on the same image dataset, which had also used to develop the segmentation algorithm. It is likely that the performance will be slightly lower when larger dataset of unseen data are considered. Secondly, the use of isotropic and small voxels (slice thickness < 5 mm) could improve the segmentation algorithm performance. In this case, in fact, it could be possible to perform a 3D segmentation and to include in the clustering approach additional spatial information (by using, for example, the hidden Markov random fields theory) [25,26]. Concerning registration method, the use of a synthetic pattern replacing tumor and necrosis ROIs, that prevents the registration algorithm from modeling fictitious deformation, had the drawback not to taking potential local deformations due to the heat coagulative effects into account. In a recent study [27], it was found that RF and microwave ablation both cause significant contraction of normal bovine liver and lung tissue ex vivo. Ablation-induced contraction appears to be tissue type and ablation modality specific. This phenomenon should be studied in detail and modeled into the registration process.

Nevertheless, this work represents a first attempt to obtain a quantitative tool aimed to assess the accuracy of RFA treatment, and its major contribution is the definition of several numerical indexes that could be helpful to quantitatively describe RFA treatment, pointing out potential limitations.

Finally, from a clinical point of view it would have been also very interesting to follow up the cases with incomplete tumor necrosis. Unfortunately we have a limited dataset consisting of 10 tumors only (5 HCC and 5 metastasis) almost heterogeneous in nature. For this purpose we are planning a new study including a larger dataset grouped for type of tumors. In this new study, we think to investigate other valuable indexes such as the largest axial diameter of tumor and necrosis and difference of the HU-units in order to provide possible surrogates for the modified RECIST criteria.

## Competing interests

The authors declare that they have no competing interests.

## Authors’ contributions

KP conceived of the study, designed the study, coordinate the image processing work and drafted the manuscript. SS participated in the design of the study and carried out the image processing and contributed in the manuscript draft. DS and DV participated in the design of the study, carried out all the radiological aspect of the work and contributed to the manuscript draft. FG participated in the design of the study, carried out all the radiofrequency ablation part of the work and helped to draft the manuscript. LM participated in study design and coordination and helped to draft the manuscript. All authors read and approved the final manuscript.

## Pre-publication history

The pre-publication history for this paper can be accessed here:

http://www.biomedcentral.com/1471-2342/13/3/prepub
